# Mechanism-informed neuroprotection in acute ischemic stroke treated with thrombectomy: a systematic review and meta-analysis of randomized controlled trials

**DOI:** 10.3389/fphar.2026.1843513

**Published:** 2026-06-17

**Authors:** Yuchen Wang, Jie Xu, Zhiheng Zhao, Ruizhe Yang, Xin Zhou, Zhang Yang

**Affiliations:** Department of Neurology, Affiliated Hospital of Guizhou Medical University, Guiyang, Guizhou, China

**Keywords:** acute ischemic stroke, cerebroprotection, endovascular thrombectomy, ischemia-reperfusion injury, meta-analysis, neuroprotective agents

## Abstract

**Background:**

Despite the success of endovascular thrombectomy (EVT) in restoring large-vessel patency in acute ischemic stroke (AIS), a substantial proportion of patients fail to achieve functional independence, largely due to ischemia-reperfusion injury (IRI) at the tissue level. Pharmacological neuroprotection has re-emerged as a potential strategy to mitigate these downstream injury cascades in the thrombectomy era; however, clinical evidence remains heterogeneous and mechanism-specific effects are unclear.

**Methods:**

We performed a systematic review and meta-analysis of randomized controlled trials evaluating neuroprotective pharmacotherapies administered as adjuncts to EVT in AIS. PubMed, Embase, and the Cochrane Library were searched from inception to 31 January 2026. The primary outcome was functional independence at 90 days (mRS 0–2). Random-effects models were used for pairwise meta-analysis, with an exploratory network meta-analysis (NMA) performed as a secondary, hypothesis-generating analysis.

**Results:**

Nine RCTs involving 4,420 patients were included. Adjunctive neuroprotective therapy was associated with a modest increase in functional independence compared with control (OR 1.13, 95% CI 1.01–1.28). However, most secondary efficacy outcomes did not show clear statistically significant benefits, and no significant differences were observed in mortality, symptomatic intracranial hemorrhage, or serious adverse events. Exploratory subgroup analyses suggested possible effect modification by treatment timing and reperfusion context, while the exploratory NMA indicated heterogeneous treatment rankings. These findings should be interpreted as a modest and heterogeneous efficacy signal rather than definitive evidence that neuroprotection is effective as a general therapeutic class.

**Conclusion:**

In the thrombectomy era, adjunctive pharmacological neuroprotection may provide a modest field-level signal of potential benefit without clear evidence of increased major safety risks. However, given the heterogeneity in pharmacological mechanisms, treatment timing, and reperfusion contexts, these findings should not be interpreted as definitive evidence of efficacy for neuroprotection as a general therapeutic class. Future mechanism-informed trials are needed to determine which neuroprotective approaches, if any, provide clinically meaningful benefit in AIS. This study was prospectively registered in PROSPERO (CRD420261284814).

**Systematic review registration:**

https://www.crd.york.ac.uk/PROSPERO/view/CRD420261284814, identifier PROSPERO (CRD420261284814)

## 1 Background

Stroke remains the second leading cause of death worldwide according to the Global Burden of Disease Study 2023 (GBD 2023 Causes of Death Collaborators, 2025). Among stroke subtypes, acute ischemic stroke (AIS) accounts for the majority of cases and remains a major cause of long-term disability and mortality, imposing a substantial burden on healthcare systems worldwide (Feigin et al., 2025). The pathophysiology of AIS is initiated by abrupt interruption of cerebral blood flow and involves a complex ischemic cascade characterized by excitotoxicity, oxidative stress, inflammatory responses, and blood–brain barrier (BBB) disruption, ultimately leading to irreversible neuronal injury and neurological dysfunction (Iadecola et al., 2020). Reperfusion therapies, including intravenous thrombolysis (IVT) and endovascular thrombectomy (EVT), have become the cornerstone of contemporary AIS management (Goyal et al., 2016; Sharma and Lee, 2025). These strategies are strongly recommended in contemporary international stroke guidelines (Xiong et al., 2025; Prabhakaran et al., 2026). Nevertheless, approximately one-third to one-half of patients fail to achieve functional independence despite successful recanalization, a phenomenon commonly referred to as futile reperfusion (Pensato et al., 2025; Yuan et al., 2026). Increasing evidence suggests that restoration of macrovascular patency alone does not fully prevent secondary injury associated with ischemia-reperfusion, including excitotoxic neuronal injury, oxidative stress, thrombo-inflammatory responses, and BBB disruption, all of which contribute to progressive tissue damage and neurological deterioration (Stoll and Nieswandt, 2019; Xu et al., 2024).

These pathophysiological considerations have renewed interest in pharmacological neuroprotection as an adjunct to reperfusion therapy. Neuroprotective agents (NPAs) are intended to attenuate the molecular and cellular mechanisms of ischemia-reperfusion injury (IRI), thereby preserving the ischemic penumbra and improving neurological recovery (Lyden et al., 2021). Historically, many neuroprotective strategies failed to demonstrate clear clinical benefit in large trials. However, most of those studies were conducted in the pre-thrombectomy era, when recanalization rates were limited and inconsistent. By contrast, the rapid and reliable reperfusion achieved with contemporary EVT may provide a more favorable biological context in which neuroprotective interventions can target IRI and potentially translate into measurable clinical benefit (Schneider et al., 2023; Hill et al., 2020). Accordingly, pharmacological modulation of reperfusion-associated injury pathways has re-emerged as a potentially important strategy for augmenting the benefits of EVT.

Despite growing interest in neuroprotective therapy in the reperfusion era, the available clinical evidence remains heterogeneous. Our previous study evaluated the comparative efficacy of several NPAs in a broader population of patients with AIS (Wang et al., 2024). However, an up-to-date synthesis specifically focused on EVT-treated randomized populations remains limited, particularly in light of several recently published trials and the distinct pharmacological context created by rapid mechanical reperfusion. Accordingly, the present study aimed to systematically evaluate the efficacy and safety of adjunctive NPAs in patients with AIS undergoing EVT using a systematic review and pairwise meta-analysis. In addition, because direct head-to-head comparisons among active neuroprotective agents are lacking, we performed an exploratory network meta-analysis (NMA) to provide indirect, hypothesis-generating comparisons and to support future trial prioritization.

## 2 Methods

This systematic review and meta-analysis was conducted in accordance with the Preferred Reporting Items for Systematic Reviews and Meta-Analyses (PRISMA) statement and the recommendations of the Cochrane Handbook for Systematic Reviews of Interventions (Page et al., 2021). The study protocol was prospectively registered in PROSPERO (CRD420261284814). No substantive amendments were made to the registered protocol.

### 2.1 Search strategy

Two investigators independently searched PubMed, Embase, and the Cochrane Library to identify randomized controlled trials evaluating neuroprotective agents in patients with AIS undergoing EVT. The search covered all records from database inception to 31 January 2026 and was updated before final study selection and data synthesis.

The search strategy combined controlled vocabulary terms (MeSH or Emtree) with free-text terms related to stroke, endovascular therapy, and neuroprotection. Stroke-related terms included “stroke”, “ischemic stroke”, “brain ischemia”, and “cerebral infarction”. EVT-related terms included “thrombectomy”, “endovascular therapy”, “mechanical thrombectomy”, and “stent retriever”. Neuroprotection-related terms included “neuroprotective agents”, “free radical scavengers”, “excitatory amino acid antagonists”, and specific drug names such as “nerinetide”, “edaravone”, “edaravone dexborneol”, “nelonemdaz”, “ApTOLL”, and “glyceryl trinitrate”. No language restrictions were applied. Detailed search strategies for each database are provided in Supplementary Tables S1–S3. In addition, we also searched ClinicalTrials.gov, WHO ICTRP, the EU Clinical Trials Register, and abstracts from International Stroke Conference (ISC) abstracts and European Stroke Organisation Conference (ESOC) abstracts to identify ongoing, unpublished, or recently completed trials. No additional completed eligible RCTs were identified. Detailed strategies are provided in Supplementary Table S4.

### 2.2 Eligibility criteria

Eligibility criteria were defined according to the PICO framework.

#### 2.2.1 Inclusion criteria

Studies were eligible if they met all of the following criteria.

1. Adult patients (≥18 years) with AIS undergoing EVT, including EVT alone or EVT combined with IVT.
2. Evaluation of NPAs administered as adjunctive therapy to EVT. NPAs were defined as single pharmacological compounds or biologics with clearly defined molecular targets or mechanisms of action.
3. Reporting at least one clinical outcome related to functional recovery or safety.
4. Randomized controlled trials.

#### 2.2.2 Exclusion criteria

Studies were excluded if.

1. They were observational studies, case series, reviews, editorials, or conference abstracts without sufficient data.
2. EVT was not performed or EVT-treated data could not be separately extracted.
3. Neuroprotective therapy was not evaluated.
4. Relevant outcome data could not be extracted.

### 2.3 Study selection

Two reviewers independently screened titles and abstracts to identify potentially eligible studies. The full texts of potentially relevant articles were then assessed for final inclusion according to the predefined eligibility criteria. Any disagreements were resolved through discussion or consultation with a third reviewer. The study selection process was documented using a PRISMA flow diagram.

### 2.4 Data extraction

Two investigators independently extracted data from the included studies using a standardized data collection form. Extracted information included study characteristics, patient demographics, details of neuroprotective interventions, reperfusion strategies, timing of neuroprotective therapy relative to EVT, and reported clinical outcomes. When necessary, additional information was obtained from supplementary materials or through contact with the corresponding authors. For trials with multiple intervention arms, relevant active arms were combined in accordance with the Cochrane Handbook to avoid double counting of the control group.

### 2.5 Outcomes

The primary outcome was functional independence at 90 days, defined as a modified Rankin Scale (mRS) score of 0–2. Secondary efficacy outcomes included excellent functional outcome (mRS 0–1 at 90 days), favorable functional outcome (mRS 0–3 at 90 days), early neurological improvement assessed by change in the National Institutes of Health Stroke Scale (NIHSS) score, and infarct volume measured on follow-up neuroimaging. Safety outcomes included all-cause mortality, symptomatic intracranial hemorrhage (sICH), and treatment-related serious adverse events. Subgroup and network meta-analyses were prespecified as exploratory analyses and are described in the statistical analysis section.

### 2.6 Risk of bias assessment

The methodological quality of the included randomized controlled trials was independently assessed by two reviewers using the Cochrane Risk of Bias 2 (RoB 2) tool (Sterne et al., 2019). The following domains were evaluated: bias arising from the randomization process, deviations from intended interventions, missing outcome data, measurement of the outcome, and selection of the reported result. Disagreements were resolved through discussion until consensus was reached.

### 2.7 Statistical analysis

Data were synthesized using pairwise random-effects meta-analysis to estimate pooled odds ratios (OR) with corresponding 95% confidence intervals (CI). For the primary outcome, absolute risk difference and number needed to treat (NNT) were additionally estimated using the pooled control-group event rate as the baseline risk. Statistical heterogeneity was assessed using the I^2^ statistic. Prespecified subgroup analyses were conducted according to reperfusion strategy (EVT alone vs. EVT combined with intravenous thrombolysis), neuroprotective mechanism (single-target vs. multi-target agents), and timing of neuroprotective intervention (before, during, or after EVT). Clinical and pharmacological heterogeneity was characterized by treatment timing, mechanism of action, dose/regimen, reperfusion context, geographic setting, and single-target *versus* multi-target classification. Given the limited number of trials within subgroups, these analyses were considered exploratory and intended to aid interpretation rather than establish definitive subgroup effects. Sensitivity analyses were performed by sequentially excluding individual studies to evaluate the robustness of the pooled estimates. Risk-of-bias assessments were conducted using Review Manager 5.4, and all forest plots and pairwise meta-analyses were generated using R software version 4.5.2.

In addition, an exploratory NMA was performed to compare the relative efficacy of different NPAs. Network analyses and treatment rankings were conducted using STATA MP 17.0. Treatment ranking probabilities were estimated using the surface under the cumulative ranking curve (SUCRA) (Salanti et al., 2011). The transitivity assumption underlying the NMA was evaluated by examining the similarity of clinical and methodological characteristics across included trials, including patient populations, reperfusion strategies, and outcome definitions. Because the treatment network had a star-shaped structure and lacked direct head-to-head comparisons between active interventions, formal assessment of inconsistency was not feasible. The NMA was therefore performed primarily to generate hypothesis-generating treatment rankings rather than definitive comparative effect estimates.

All statistical tests were two-sided, and P value <0.05 was considered statistically significant. To explore potential small-study effects, a comparison-adjusted funnel plot was generated for the primary outcome and interpreted as a visual, exploratory assessment only. In accordance with Cochrane recommendations, formal asymmetry testing (e.g., Egger’s test) was not performed because fewer than 10 trials were included; accordingly, assessment was limited to visual inspection.

### 2.8 Certainty of evidence assessment

The certainty of evidence for each outcome was evaluated using the Grading of Recommendations Assessment, Development and Evaluation (GRADE) framework (Guyatt et al., 2008). Evidence quality was assessed across the domains of risk of bias, inconsistency, indirectness, imprecision, and publication bias, and was categorized as high, moderate, low, or very low certainty.

## 3 Results

### 3.1 Study selection

**FIGURE 1:**
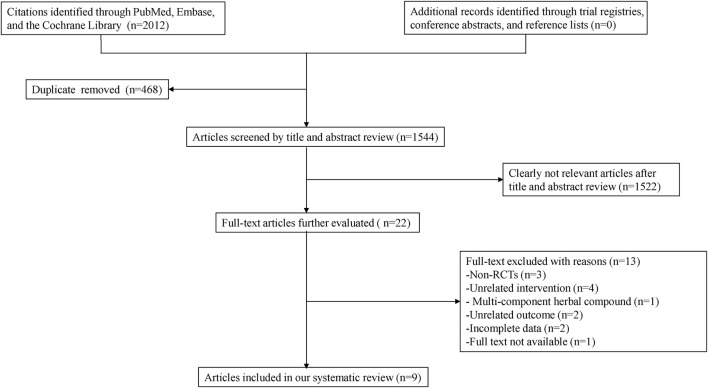
PRISMA flow diagram of study selection, including database, trial registry, conference abstract, and reference-list searches.

The study selection process is shown in Figure 1. A total of 2012 records were identified through database searching. After removal of 468 duplicates, 1544 records remained for title and abstract screening. Following screening, 22 articles were assessed in full text for eligibility. Of these, 13 studies were excluded for not meeting the predefined eligibility criteria. Ultimately, nine RCTs were included in the final meta-analysis. Supplementary searches of trial registries, conference abstracts, and reference lists did not identify any additional completed RCTs meeting the predefined eligibility criteria. The detailed PRISMA flow diagram of study selection is shown in Figure 1.

### 3.2 Characteristics of the included studies

Nine RCTs published between 2020 and 2026 were included, comprising 4420 patients with acute ischemic stroke undergoing EVT. The neuroprotective agents evaluated were edaravone dexborneol (ED; 2 trials), nerinetide (also known as NA-1; 2 trials), nelonemdaz (2 trials), ApTOLL (1 trial), glyceryl trinitrate (GTN; 1 trial), and RNS60 (1 trial) (Hill et al., 2020; Hong et al., 2022; Cheng et al., 2023; Hernández-Jiménez et al., 2023; Chen et al., 2025; Ghosh et al., 2025; Hill et al., 2025; Lee et al., 2025; Wang et al., 2026). Most studies were conducted in Asia, including three from China and two from South Korea, whereas the remaining studies were conducted in North America and Europe.

All included trials compared neuroprotective therapy with placebo or standard care in patients undergoing EVT. The timing of neuroprotective administration varied across studies and included before, during, or after EVT. Detailed baseline characteristics of the included studies are summarized in Table 1.

**TABLE 1:** Characteristics of the randomized controlled trials included in the meta-analysis.

Study	Country	Sample size (E/C)	Intervention protocol (E/C)	Age (year)	Dose and administration	Reperfusion Therapy	Timing category	Target category
ESCAPE-NA1 (2020)	Canada	549/556	NA-1**/**Placebo	71.5 (61.1–79.7)**/**70.3 (60.4–80.1)	2.6 mg/kg (max 270 mg), IV single dose	EVT/EVT+IVT	During EVT	Single-pathway
ESCAPE-NEXT (2025)	Canada	454/398	NA-1**/**Placebo	75 (65–83)**/**76 (66–83)	2.6 mg/kg (max 270 mg), IV single dose	EVT	During EVT	Single-pathway
SONIC (2022)	South Korea	122/61	Nelonemdaz (combined doses)**/**Placebo	68.6 ± 11.0**/**64.9 ± 13.3**/**70.0 ± 10.1	750 mg loading +250–500 mg q12h IV	EVT + IVT	Before EVT	Multi-target
RODIN (2025)	South Korea	232/225	Nelonemdaz**/**Placebo	72.3 ± 12.4**/**73.4 ± 11.9	750 mg loading +250–500 mg q12h IV	EVT	During EVT	Multi-target
April (2023)	Spain and France	84/55	ApTOLL (combined doses)**/**Placebo	71.29 ± 10.97**/**68.33 ± 12.51**/**71.13 ± 12.86	0.2 mg/kg IV single dose	EVT/EVT+IVT	After EVT	Single-pathway
AGAIN (2023)	China	20/20	GTN**/**Placebo	65.9 ± 7.4**/**64.5 ± 14.1	0.8 mg intra-arterial single dose	EVT	During EVT	Single-pathway
INSIST-ED (2025)	China	97/103	ED**/**Placebo	63.61 ± 10.75**/**63.31 ± 10.84	37.5 mg IV twice daily for 12 days	EVT	After EVT	Multi-target
TASTE-2 (2026)	China	690/672	ED**/**Placebo	67 (57–73)**/**67(58–73)	37.5 mg IV twice daily for 10–14 days	EVT	During EVT	Multi-target
RESCUE (2025)	USA	54/28	RNS60 (combined doses)**/**Placebo	68.0 ± 12.5**/**67.8 ± 10.7**/**66.0 ± 12.5	0.5–1.0 mL/kg/h IV infusion	EVT	Before EVT	Single-pathway

Abbreviations: E/C, experimental group/control group; EVT, endovascular therapy; IVT, intravenous thrombolysis; NA-1, nerinetide; GTN, glyceryl trinitrate; ED, edaravone dexborneol. Values for age are presented as mean ± standard deviation (SD) or median (interquartile range) as reported in the original trials. For trials with multiple intervention arms (e.g., SONIC, April, and RESCUE), intervention arms were combined for the primary meta-analysis according to the recommendations of the Cochrane Handbook. “EVT/EVT + IVT” indicates that all analyzed patients underwent EVT, with some also receiving bridging intravenous thrombolysis. IVT-only populations were not included in the quantitative synthesis.

### 3.3 Risk of bias assessment

**FIGURE 2:**
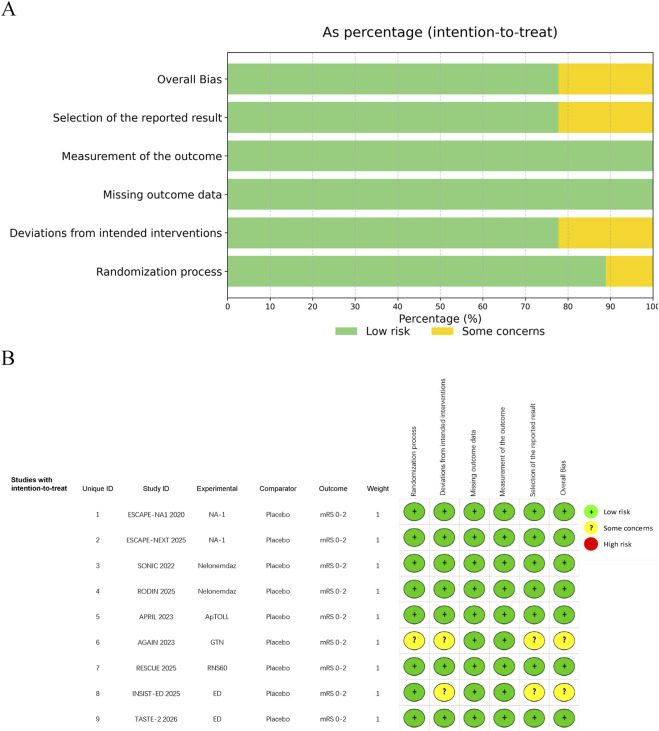
Risk of bias assessment using the Cochrane Risk of Bias 2 (RoB 2) tool for the primary outcome (functional independence, mRS 0–2 at 90 days). **(A)** Overall risk-of-bias summary presented as percentages under the intention-to-treat framework across all included randomized controlled trials, showing the distribution of “low risk” and “some concerns” across bias domains. **(B)** Domain-level risk-of-bias assessment for individual studies, including bias arising from the randomization process, deviations from intended interventions, missing outcome data, measurement of the outcome, and selection of the reported result. Green indicates low risk of bias, yellow indicates some concerns, and no high-risk judgments were identified.

The overall methodological quality of the included trials was generally high according to the RoB 2 assessment. Most studies were judged to have low risk of bias across the domains of the randomization process, deviations from intended interventions, missing outcome data, measurement of the outcome, and selection of the reported result. Some concerns were identified in a small number of studies, mainly in the domains of the randomization process, deviations from intended interventions, or selection of the reported result. No included study was judged to be at high risk of bias. A graphical summary of the RoB 2 assessment is presented in Figure 2.

### 3.4 Primary outcome

**FIGURE 3:**
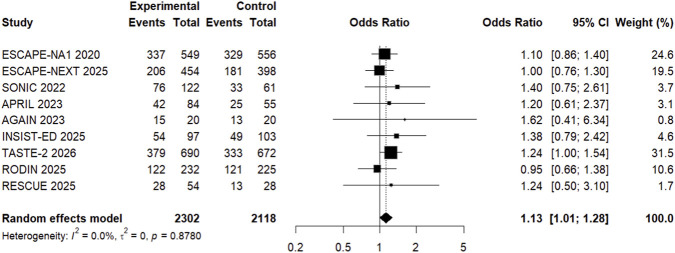
Forest plot of the primary outcome: functional independence at 90 days (mRS 0–2). The pooled estimate compares neuroprotective therapy with control treatment in patients undergoing EVT. Odds ratios with 95% confidence intervals (CI) were calculated using a random-effects model.

All nine included trials reported the primary outcome of functional independence at 90 days, defined as a mRS score of 0–2. Pooled analysis showed that adjunctive neuroprotective therapy was associated with a significantly higher likelihood of achieving functional independence than control treatment (OR 1.13, 95% CI 1.01–1.28; *p* = 0.03). Based on the pooled control-group event rate, this corresponded to an absolute increase of approximately 30 additional patients achieving functional independence per 1000 treated patients, with an estimated NNT of approximately 33. The corresponding forest plot is shown in Figure 3.

### 3.5 Secondary outcomes

Secondary outcomes included excellent functional outcome (mRS 0–1 at 90 days), favorable functional outcome (mRS 0–3 at 90 days), early neurological improvement, and infarct volume.

For excellent functional outcome and favorable functional outcome, pooled analyses did not show statistically significant differences between the neuroprotective therapy and control groups. For early neurological improvement, assessed by change in NIHSS score, the pooled estimate suggested a numerical benefit with neuroprotective therapy. For infarct volume, pooled analysis indicated a small reduction, although the result was not statistically significant and varied across studies. Detailed results for secondary efficacy outcomes are presented in Supplementary Figure S1. Overall, these secondary efficacy outcomes did not provide consistent statistically significant support for the primary outcome finding and should therefore be interpreted as exploratory signals rather than confirmatory evidence of broad clinical efficacy.

### 3.6 Safety outcomes

**FIGURE 4:**
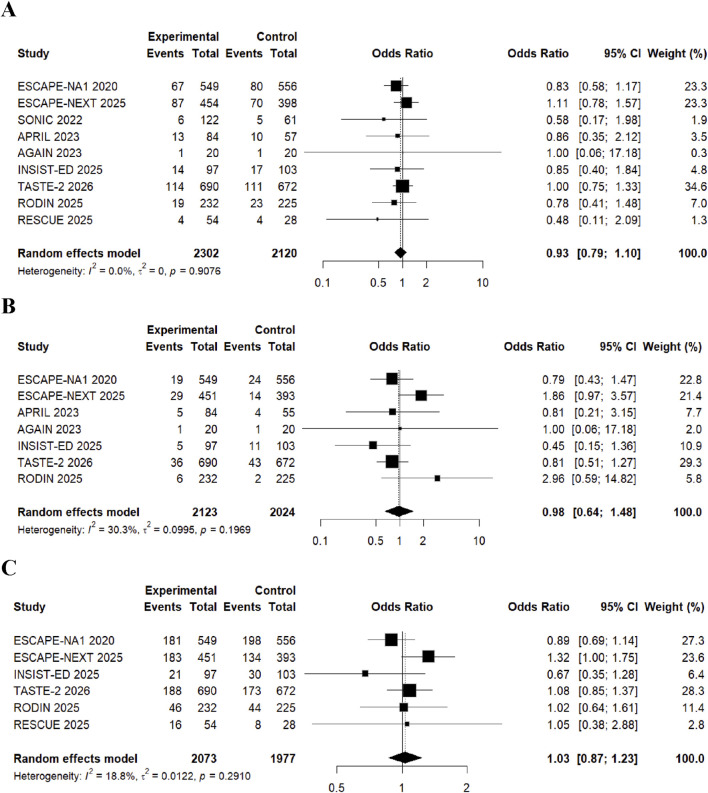
Forest plots of safety outcomes. **(A)** All-cause or stroke-related mortality. **(B)** Symptomatic intracranial hemorrhage (sICH). **(C)** Other treatment-related serious adverse events.

Safety outcomes included all-cause mortality, symptomatic intracranial hemorrhage (sICH), and serious adverse events. Pooled analyses showed no statistically significant differences between neuroprotective therapy and control treatment for mortality, sICH, or serious adverse events. The corresponding forest plots are shown in Figure 4.

### 3.7 Subgroup analysis

Prespecified subgroup analyses were conducted according to reperfusion strategy (EVT alone vs. EVT plus intravenous thrombolysis), neuroprotective mechanism (single-target vs. multi-target agents), and timing of neuroprotective intervention (before, during, or after EVT).

**FIGURE 5:**
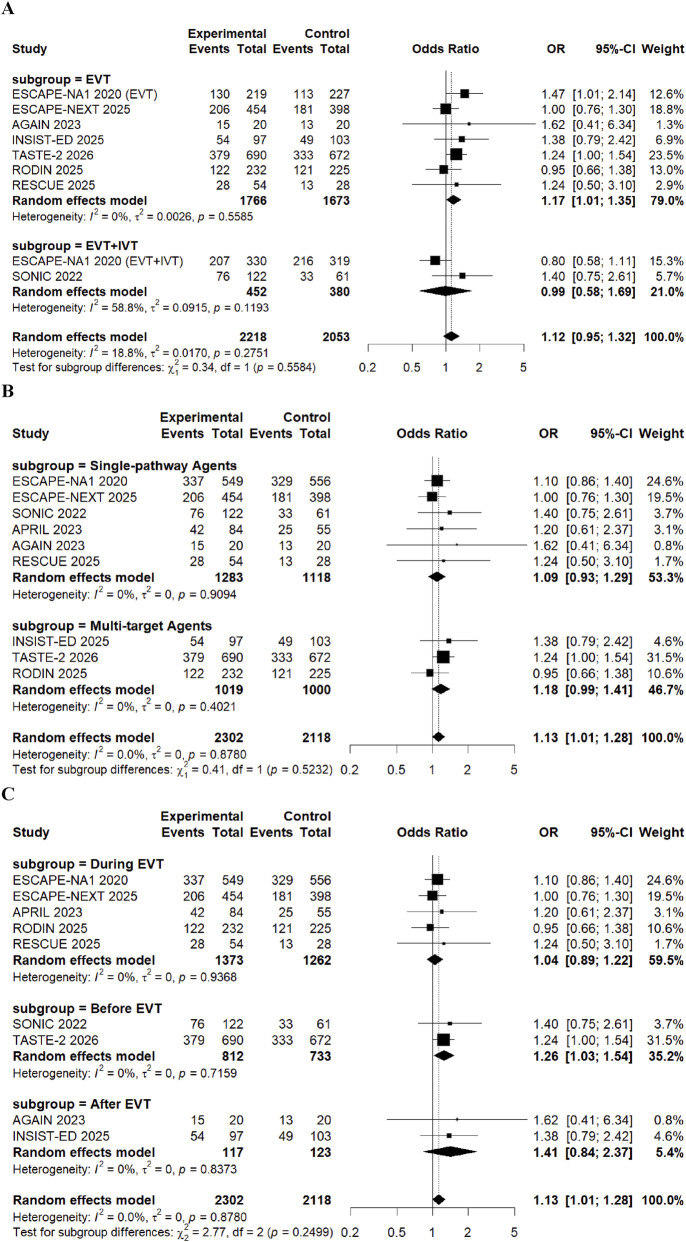
Subgroup analyses of functional independence at 90 days (mRS 0–2). **(A)** Subgroup analysis according to reperfusion strategy. **(B)** Subgroup analysis according to neuroprotective mechanism. **(C)** Subgroup analysis according to timing of neuroprotective intervention. These subgroup analyses were hypothesis-generating and should not be interpreted as confirmatory evidence of differential treatment effects.

The effect estimate appeared numerically larger among patients treated with EVT alone than among those receiving EVT combined with intravenous thrombolysis. Multi-target agents also showed numerically greater treatment effects than single-target agents, and administration before EVT was associated with a trend toward improved functional outcomes. However, these subgroup findings were based on a limited number of trials and should be interpreted as exploratory and hypothesis-generating. Detailed subgroup results are shown in Figure 5.

### 3.8 Publication bias

Visual inspection of the comparison-adjusted funnel plot for the primary outcome showed general symmetry, with no obvious evidence of publication bias or small-study effects. However, because the number of included trials was limited (n = 9), this assessment should be interpreted cautiously. The funnel plot is presented in Supplementary Figure S2.

### 3.9 Sensitivity analysis

Sensitivity analyses were performed by sequentially excluding individual studies from the primary outcome analysis. The pooled effect estimate remained materially unchanged after omission of each study, indicating that the primary result was not driven by any single trial. Detailed results are shown in Supplementary Figure S3.

### 3.10 Exploratory network meta-analysis of different neuroprotective agents

**FIGURE 6:**
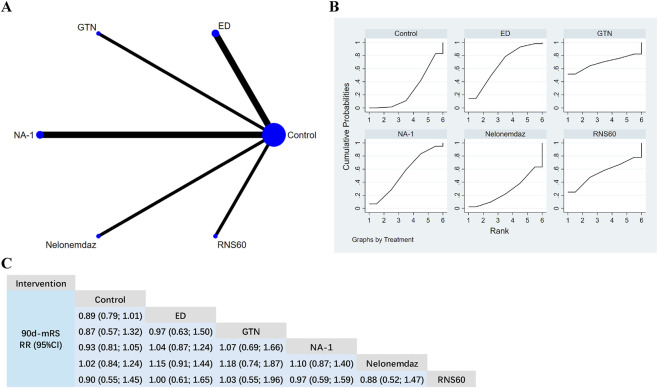
Exploratory network meta-analysis of functional independence (mRS 0–2 at 90 days). **(A)** Network plot of the included interventions. Node size reflects the number of participants, and line thickness indicates the number of studies for each comparison. **(B)** Cumulative ranking probability curves based on SUCRA values. **(C)** League table showing relative treatment effects between interventions. Because the network was star-shaped and lacked direct active-to-active comparisons, SUCRA rankings should be interpreted as hypothesis-generating rather than comparative evidence for clinical decision-making.

An exploratory network meta-analysis was conducted to compare the relative efficacy of different neuroprotective agents. The treatment network had a star-shaped geometry, with all active interventions compared only with control treatment and no direct head-to-head comparisons between active agents. Based on SUCRA ranking probabilities, GTN and ED ranked among the most favorable interventions for improving functional independence. However, because the network had a star-shaped structure and lacked direct active-to-active comparisons, these rankings should be interpreted only as exploratory and hypothesis-generating. They should not be considered comparative evidence of treatment superiority or used to guide clinical decision-making. The network structure and relative treatment effects are shown in Figure 6, and detailed ranking probabilities and cumulative ranking curves are presented in Supplementary Figure S4.

### 3.11 Certainty of evidence (GRADE)

The certainty of evidence for each outcome was assessed using the GRADE framework (Table 2). The evidence for the primary outcome, functional independence at 90 days, was rated as moderate certainty. Most secondary functional and safety outcomes were also rated as moderate certainty, mainly because of imprecision in the effect estimates. The certainty of evidence for infarct volume was rated as low because of the limited number of available studies and heterogeneity in outcome assessment. Overall, the evidence suggests a modest efficacy signal for adjunctive neuroprotective therapy in EVT-treated patients, with no clear increase in major safety risks, although the certainty of evidence varied across outcomes.

**TABLE 2:** Summary of findings and certainty of evidence (GRADE).

Outcome	Participants (RCTs)	Relative effect (95% CI)	Absolute effect*	Certainty of evidence	Main GRADE considerations
mRS 0–2 at 90 days (primary outcome)	4420 (9)	OR 1.13 (1.01–1.28)	30 more per 1000 (2–61 more); NNT ≈33	⊕⊕⊕⃝Moderate	Imprecision: CI close to null; modest absolute benefit
mRS 0–1 at 90 days	4380 (8)	OR 1.14 (0.97–1.34)	18 more per 1000 (16 fewer to 59 more)	⊕⊕⊕⃝Moderate	Imprecision: CI included no effect
mRS 0–3 at 90 days	4420 (9)	OR 1.08 (0.95–1.22)	14 more per 1000 (8 fewer to 34 more)	⊕⊕⊕⃝Moderate	Imprecision: CI included no effect; small absolute effect
Change in NIHSS score	1541 (3)	MD −0.49 (−1.22 to 0.25)	0.49-Point greater improvement	⊕⊕⊕⃝Moderate	Imprecision: Limited trials; CI crossed no effect
Infarct volume	1905 (4)	SMD −0.22 (−0.85 to 0.40)	Small reduction in infarct volume; not statistically significant	⊕⊕⃝⃝Low	Imprecision and inconsistency: wide CI; variable assessment across trials
All-cause mortality	4422 (9)	OR 0.92 (0.78–1.09)	12 fewer per 1000 (31 fewer to 13 more)	⊕⊕⊕⃝Moderate	Imprecision: CI included potential benefit and harm
Symptomatic ICH	4147 (7)	OR 1.02 (0.77–1.36)	1 more per 1000 (11 fewer to 18 more)	⊕⊕⊕⃝Moderate	Imprecision: CI included potential benefit and harm
Serious adverse events	4050 (6)	OR 1.04 (0.78–1.39)	10 more per 1000 (20 fewer to 42 more)	⊕⊕⊕⃝Moderate	Imprecision: CI included potential benefit and harm

Abbreviations: AIS, acute ischemic stroke; CI, confidence interval; EVT, endovascular therapy; GRADE, grading of recommendations assessm ent, Development and Evaluation; ICH, intracranial hemorrhage; MD, mean difference; mRS, modified Rankin Scale; NIHSS, national institutes of health stroke scale; NNT, number needed to treat; NS, not statistically significant; OR, odds ratio; RCTs, randomized controlled trials; SMD, standardized mean difference.

* Absolute effects were calculated using the pooled odds ratios and the pooled control-group event rate as the baseline risk. For the primary outcome, the number needed to treat was estimated from the absolute risk difference. GRADE, considerations summarize the main reasons for downgrading. Across outcomes, certainty was not downgraded for risk of bias, indirectness, or publication bias unless otherwise specified. Inconsistency was downgraded only for infarct volume because outcome assessment varied across trials.

## 4 Discussion

### 4.1 Summary of main findings

In this systematic review and meta-analysis of nine RCTs involving 4420 patients with AIS treated with EVT, adjunctive neuroprotective pharmacotherapy was associated with a statistically significant increase in functional independence at 90 days compared with control treatment. No clear increase was observed in major safety outcomes, including mortality, symptomatic intracranial hemorrhage, and serious adverse events. These findings suggest a modest and heterogeneous field-level signal for adjunctive pharmacological neuroprotection in the EVT era, rather than definitive evidence that neuroprotection is effective as a general therapeutic class. However, the magnitude of benefit was small, and the confidence interval was close to the null. Therefore, the primary result should be interpreted cautiously, particularly because most secondary efficacy outcomes, including excellent functional outcome, favorable functional outcome, early neurological improvement, and infarct volume, did not show clear statistically significant benefits. Rather than establishing broad or uniform clinical efficacy of neuroprotection, the present findings should be viewed as a modest and heterogeneous efficacy signal that supports further investigation of selected mechanism-informed strategies in appropriately defined EVT-treated populations.

Our findings are broadly consistent with recent evidence syntheses evaluating neuroprotective strategies in the reperfusion era. A recent meta-analysis of randomized trials assessing neuroprotective agents administered alongside reperfusion therapies also reported an increased likelihood of functional independence at 90 days, with the treatment signal appearing more pronounced among patients undergoing EVT (Wesley et al., 2025). Compared with Zhang et al. (Zhang et al., 2026), who evaluated broader reperfusion populations including IVT, EVT, and IVT plus EVT, our review specifically focused on randomized EVT-treated patients and updated the evidence base to 31 January 2026. Although the number of EVT-relevant trials was similar, the study composition differed because of differences in eligibility criteria, handling of mixed reperfusion populations, and inclusion of newer EVT-era RCTs. Regarding nerinetide, our review included the two EVT-focused RCTs that met our prespecified eligibility criteria. FRONTIER was a prehospital suspected-stroke trial rather than an EVT-treated AIS trial, and its EVT findings were based on exploratory post-randomization subgroup analyses (Christenson et al., 2025). The subsequent *post hoc* individual patient data meta-analysis pooled early-window patients selected for reperfusion with thrombolysis, EVT, or both (Tymianski et al., 2025). Therefore, these studies addressed a different question and were discussed for context but not pooled in our EVT-treated RCT-level quantitative synthesis. Similarly, the updated edaravone-dexborneol meta-analysis by Sabet et al. evaluated a broader Chinese AIS population and included both randomized and observational studies, whereas our ED evidence was restricted to EVT-treated RCTs (Sabet et al., 2026). Thus, previous ED-specific evidence supports the potential neuroprotective role of edaravone-dexborneol in AIS, while the present study specifically addresses its role within an EVT-treated, RCT-level, multi-agent framework.

**FIGURE 7:**
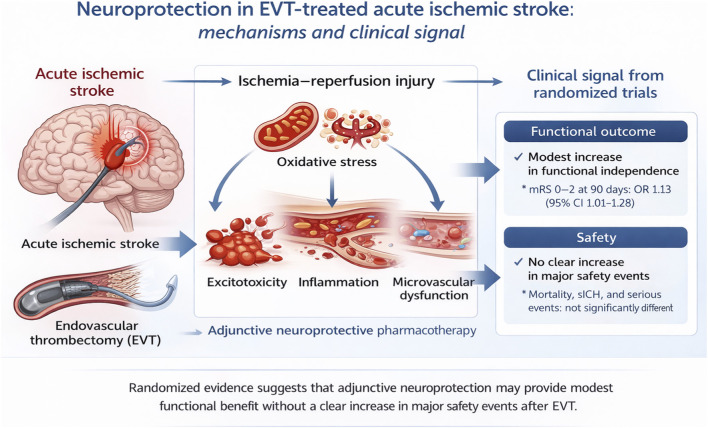
Neuroprotection in EVT-treated acute ischemic stroke: mechanisms and clinical implications. IRI after EVT involves oxidative stress, excitotoxicity, inflammation, and microvascular dysfunction. Adjunctive neuroprotective therapies target these pathways. Randomized evidence indicates a modest improvement in functional independence (mRS 0–2 at 90 days; OR 1.13, 95% CI 1.01–1.28) without a significant increase in major safety events. This schematic links key pathophysiological mechanisms with clinical outcomes.

Taken together, these findings suggest that adjunctive neuroprotection may provide modest benefit in EVT-treated acute ischemic stroke, with effects likely to be mechanism-specific rather than a uniform class effect. Because macrovascular recanalization does not fully prevent tissue-level ischemia–reperfusion injury after thrombectomy, we provide a schematic overview linking major pathophysiological pathways with the overall clinical signal observed across randomized trials (Figure 7).

Clinical and pharmacological heterogeneity across the included trials should also be considered when interpreting the pooled estimates. The evaluated agents differed in mechanism of action, timing of administration relative to EVT, dose and regimen, reperfusion context, and geographic setting. In particular, EVT alone *versus* EVT combined with IVT may be relevant for agents susceptible to pharmacological interaction with thrombolytic therapy. In addition, single-target and multi-target agents may not be biologically equivalent, given the multifactorial nature of ischemia-reperfusion injury. These factors support a cautious interpretation of the pooled class-level estimate and reinforce that adjunctive neuroprotection after EVT should be viewed as context-dependent and mechanism-specific rather than as a uniform therapeutic class effect.

### 4.2 Biological and pharmacological interpretation

The present findings should be interpreted in the context of the evolving pathophysiological understanding of IRI in the thrombectomy era. EVT has substantially improved recanalization rates and clinical outcomes in AIS, yet a considerable proportion of patients still fail to achieve favorable recovery despite successful reperfusion, a phenomenon often referred to as futile reperfusion (Elmadhoun et al., 2024). This observation highlights an important therapeutic gap between restoration of macrovascular patency and tissue-level recovery.

Experimental and translational studies have shown that reperfusion does not terminate ischemic injury, but instead may trigger or amplify a complex cascade involving excitotoxicity, oxidative stress, mitochondrial dysfunction, neuroinflammation, microvascular failure, and BBB disruption (Soldozy et al., 2023; Bai et al., 2025; Kovacheva et al., 2025). Accordingly, restoration of large-vessel flow alone may be insufficient to prevent ongoing cellular and microvascular injury. This provides a strong biological rationale for combining reperfusion therapies with pharmacological interventions targeting downstream injury pathways.

From a pharmacological perspective, neuroprotective therapy should not be viewed as a single therapeutic category, but rather as a heterogeneous group of interventions acting on distinct components of ischemic brain injury (Zhao et al., 2024). The NPAs included in the present analysis illustrate this mechanistic diversity. Edaravone dexborneol (ED) combines free radical scavenging with anti-inflammatory activity, thereby targeting oxidative and inflammatory components of reperfusion injury simultaneously (Xu et al., 2021; Fu et al., 2024). Nerinetide and nelonemdaz both aim to attenuate excitotoxic injury, although through different pharmacological mechanisms: nerinetide disrupts the interaction between the N-methyl-D-aspartate receptor (NMDAR) and postsynaptic density protein-95 (PSD-95), whereas nelonemdaz acts as an NMDAR antagonist (Christenson et al., 2025; Tymianski et al., 2025; Zeng et al., 2026). ApTOLL targets Toll-like receptor 4-mediated innate immune activation and therefore represents a more inflammation-oriented strategy (Hernández-Jiménez et al., 2022). Glyceryl trinitrate (GTN) may improve collateral or microvascular perfusion through nitric oxide-mediated vasodilation (Jiang et al., 2024), whereas RNS60 has been linked to modulation of neuroinflammatory and bioenergetic pathways (Baena-Caldas et al., 2024). Taken together, these agents underscore that the clinical concept of neuroprotection in stroke is fundamentally multi-mechanistic.

Importantly, the EVT setting may provide a particularly relevant therapeutic context for such interventions. Rapid and reliable reperfusion increases the likelihood that salvageable tissue remains present at the time of treatment, while simultaneously exposing that tissue to reperfusion-associated injury processes (Cheng et al., 2024). This therapeutic context may help explain why neuroprotective approaches that showed limited benefit in the pre-thrombectomy era warrant renewed evaluation in contemporary practice. In this respect, the present findings are consistent with the STAIR XI framework, which advocates a shift from single-pathway neuroprotection toward integrated cerebroprotective strategies used in conjunction with reperfusion therapy (Lyden et al., 2021). Therefore, the modest overall benefit observed in our analysis should be interpreted as a broad field-level signal rather than evidence of a generalized neuroprotective class effect. The available data suggest that potential benefit is likely to depend on the interaction between drug mechanism, biological timing, concomitant reperfusion therapy, and patient selection. This distinction is important because pooling pharmacologically heterogeneous agents may obscure meaningful differences between individual interventions. The principal pharmacological targets, mechanisms of action, and stages of ischemic injury addressed by the included agents are summarized in Table 3.

**TABLE 3:** Pharmacological targets and mechanisms of neuroprotective agents.

Neuroprotective agent	Pharmacological target	Mechanism of action	Stage of ischemic injury targeted
Edaravone dexborneol	Oxidative stress and inflammatory pathways	Combined free radical scavenging and anti-inflammatory activity, reducing lipid peroxidation and inflammatory signaling	Ischemia-reperfusion injury
Nerinetide	PSD-95/NMDA receptor signaling	Disrupts the NMDA receptor–PSD-95 interaction, thereby attenuating excitotoxic neuronal injury	Early excitotoxic phase
Nelonemdaz	NMDA receptor antagonist	Inhibits NMDA receptor–mediated calcium influx and subsequent excitotoxic neuronal damage	Early excitotoxic phase
ApTOLL	Toll-like receptor 4 (TLR4) signaling	Inhibits TLR4-mediated innate immune activation and downstream inflammatory responses after ischemia	Neuroinflammatory phase
Glyceryl trinitrate	Nitric oxide signaling	Promotes nitric oxide–mediated vasodilation and may enhance collateral circulation and microvascular perfusion	Microvascular dysfunction
RNS60	Cellular bioenergetics and anti-inflammatory pathways	Modulates mitochondrial bioenergetics and attenuates inflammatory responses	Mitochondrial dysfunction and inflammation

Abbreviations: NMDA, N-methyl-D-aspartate; PSD-95, postsynaptic density protein-95; TLR4, Toll-like receptor 4. The table summarizes the principal pharmacological targets and mechanisms of neuroprotective agents evaluated in the included randomized controlled trials. Mechanistic descriptions are based on evidence from preclinical and clinical pharmacological studies of ischemia-reperfusion injury.

### 4.3 Interpretation of subgroup effects

The subgroup analyses provide exploratory insights into factors that may influence the therapeutic effects of adjunctive NPAs. The observed trend toward greater benefit when neuroprotective therapy was initiated before EVT is biologically plausible, as earlier intervention may help stabilize vulnerable tissue before reperfusion occurs and attenuate the earliest phases of reperfusion-associated injury. However, because these analyses were based on a limited number of trials within each subgroup and were performed at the study level, they should be interpreted cautiously and regarded as hypothesis-generating rather than confirmatory evidence.

An additional clinically relevant observation relates to differences in reperfusion strategy. In the ESCAPE-NA1 trial, the apparent divergence in treatment effects between patients treated with EVT alone and those receiving EVT combined with intravenous thrombolysis suggested that the efficacy of some neuroprotective agents may be modified by concomitant therapies (Hill et al., 2020). One plausible explanation is pharmacological interaction. Mechanistic studies have demonstrated that alteplase-generated plasmin can cleave nerinetide and reduce its biological activity, providing a concrete example of how drug–drug interactions may alter neuroprotective efficacy in the reperfusion setting (Mayor-Nunez et al., 2021). This observation has important implications for future trial design, as pharmacological neuroprotection may need to be evaluated not only according to molecular mechanism and timing, but also according to the broader reperfusion strategy in which the agent is deployed.

The numerically greater effects observed with multi-target agents are also noteworthy. Because IRI is multifactorial and evolves across several overlapping biological stages, interventions that affect more than one injury pathway may be more likely to confer clinically measurable benefit than agents directed at a single target (Zhao et al., 2024). Emerging clinical data are compatible with this view. A secondary analysis of the MARVEL randomized clinical trial suggested that adjunctive methylprednisolone may improve functional outcomes in patients with acute internal carotid artery occlusion undergoing thrombectomy (Zheng et al., 2025). In addition, pooled data from the RESCUE-BT and MARVEL trials suggested that combined tirofiban and methylprednisolone therapy, targeting thrombotic and inflammatory pathways simultaneously, may improve post-EVT outcomes (Ye et al., 2026). Although these observations should not be overinterpreted, they are consistent with the broader concept that mechanism-informed, multi-pathway adjunctive strategies may represent a productive direction for future cerebroprotection research.

### 4.4 Network meta-analysis interpretation and translational implications

The exploratory network meta-analysis (NMA) was undertaken to provide an indirect comparison of different neuroprotective agents in the absence of head-to-head trials. According to SUCRA ranking probabilities, GTN and ED ranked among the more favorable interventions for improving functional independence. However, because the treatment network had a star-shaped structure and all active interventions were connected only through control arms, these rankings were driven entirely by indirect comparisons. Therefore, SUCRA rankings should not be interpreted as evidence of superiority between active agents or used for comparative clinical decision-making. The NMA should be regarded primarily as a hypothesis-generating framework for future trial prioritization.

Even so, the exploratory NMA remains informative in several respects. First, it provides a structured overview of the comparative landscape of candidate neuroprotective agents in EVT-treated AIS. Second, it highlights the possibility that efficacy may differ across mechanistic classes, rather than being shared uniformly across all neuroprotective approaches. Third, it may help identify agents or mechanistic strategies that warrant prioritization in future confirmatory trials. Rather than establishing superiority of any individual intervention, the present NMA should therefore be viewed primarily as a tool for hypothesis generation and trial prioritization (Florez et al., 2024).

From a translational perspective, the present findings support the concept that pharmacological neuroprotection may serve as a bridge between successful recanalization and tissue-level recovery (Menzies et al., 2025). EVT restores macrovascular flow, but it does not directly address the molecular and cellular injury processes that continue after reperfusion. Agents targeting excitotoxicity, oxidative stress, neuroinflammation, and microvascular dysfunction may therefore complement EVT by reducing secondary injury in reperfused but vulnerable tissue. Future translational and clinical research should focus not only on whether neuroprotection works, but also on which mechanisms, timing strategies, and reperfusion contexts are most likely to yield meaningful clinical benefit.

### 4.5 Strengths and limitations

This study has several strengths. First, only RCTs were included, which improves the internal validity of the pooled estimates and reduces the risk of confounding commonly encountered in non-randomized evidence. Second, the review was conducted according to PRISMA principles, prospectively registered, and supplemented by risk-of-bias and GRADE assessments, thereby enhancing methodological transparency and interpretability. Third, by focusing specifically on EVT-treated populations, the present analysis addresses a clinically important and biologically distinct treatment context in which neuroprotective strategies may be more relevant than in the pre-thrombectomy era. Finally, the inclusion of an exploratory NMA allowed a structured, mechanism-oriented overview of the comparative landscape of candidate NPAs, while acknowledging the limitations of indirect evidence.

Several limitations should also be acknowledged. First, although all included studies were randomized trials, the total number of available studies remained modest, and the sample sizes for several individual agents were limited. Second, the overall treatment effect for the primary outcome was statistically significant but modest in magnitude, and its clinical implications should therefore be interpreted with appropriate caution. Third, the included NPAs differed substantially in mechanism of action, dose and regimen, timing of administration, geographic setting, and reperfusion context, including EVT alone *versus* EVT combined with IVT. In addition, single-target and multi-target agents were pooled in the overall class-level analysis despite their biological differences. These factors introduce unavoidable clinical and pharmacological heterogeneity and limit the extent to which the pooled estimate can be interpreted as evidence of a uniform neuroprotective class effect. Fourth, several secondary outcomes were reported in only a small number of trials, which reduces confidence in those estimates. Fifth, the exploratory NMA was based on a star-shaped network without closed loops, precluding formal assessment of inconsistency and limiting the robustness of indirect comparisons between active interventions. Finally, pooled class-level estimates may obscure important differences between individual agents, and the present findings should therefore not be interpreted as evidence that all neuroprotective strategies are equally effective in EVT-treated acute ischemic stroke. Although trial registries and selected gray literature sources were searched, unpublished or conference-only studies may still have been missed. Therefore, residual publication bias cannot be fully excluded. Despite these limitations, the available evidence supports continued investigation of mechanism-informed neuroprotective strategies as adjuncts to EVT.

## 5 Conclusion

Adjunctive pharmacological neuroprotection may be associated with a modest and heterogeneous signal of clinical benefit in patients with AIS undergoing EVT, without clear evidence of increased major safety risks. However, these findings should not be interpreted as definitive evidence that neuroprotection is effective as a general therapeutic class. In the thrombectomy era, neuroprotective therapy may be better conceptualized as a context-dependent and mechanism-specific strategy aimed at addressing the gap between successful macrovascular recanalization and tissue-level recovery. Future research should prioritize mechanism-informed trial designs that integrate pharmacological target, timing of administration, reperfusion context, and potential drug–drug interactions to identify which strategies, if any, provide clinically meaningful benefit.

## Data Availability

The original contributions presented in the study are included in the article/Supplementary Material, further inquiries can be directed to the corresponding author.
