# Supplementary material for: Mechanism-informed neuroprotection in acute ischemic stroke treated with thrombectomy: a systematic review and meta-analysis of randomized controlled trials
Source: Front Pharmacol. 2026 Jun 17;17:1843513. doi: 10.3389/fphar.2026.1843513 (PMC13318870; doi:10.3389/fphar.2026.1843513)
Supplement: Supplementary file 1 [file Supplementaryfile1.docx]

Supplementary Material


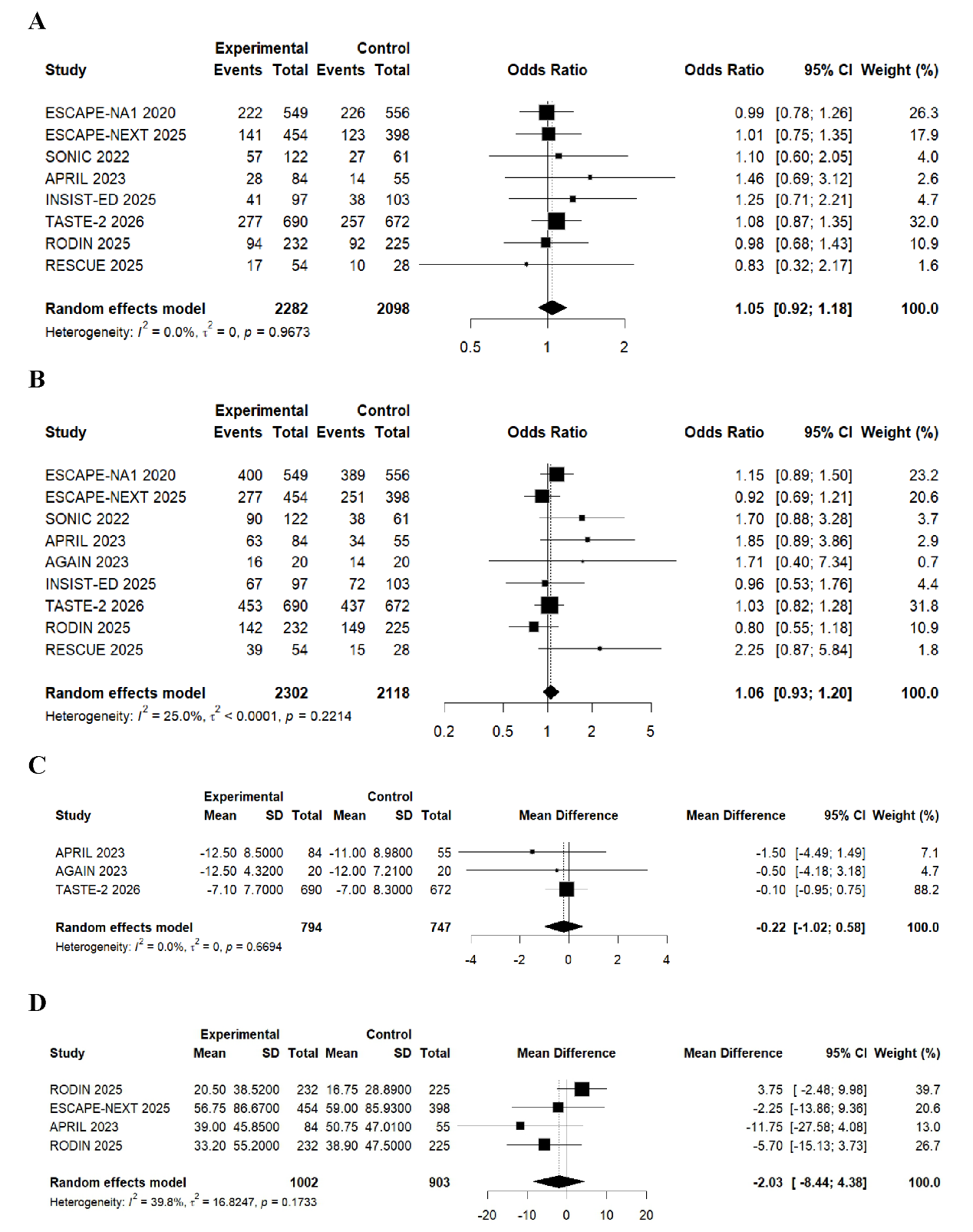
**1. Supplementary Figures**

**Supplementary Figure S1.** Forest plots of secondary efficacy outcomes. (A) Excellent functional outcome (mRS 0–1 at 90 days). (B) Favorable functional outcome (mRS 0–3 at 90 days). (C) Early neurological improvement based on changes in NIHSS score. (D) Infarct volume measured on follow-up neuroimaging.


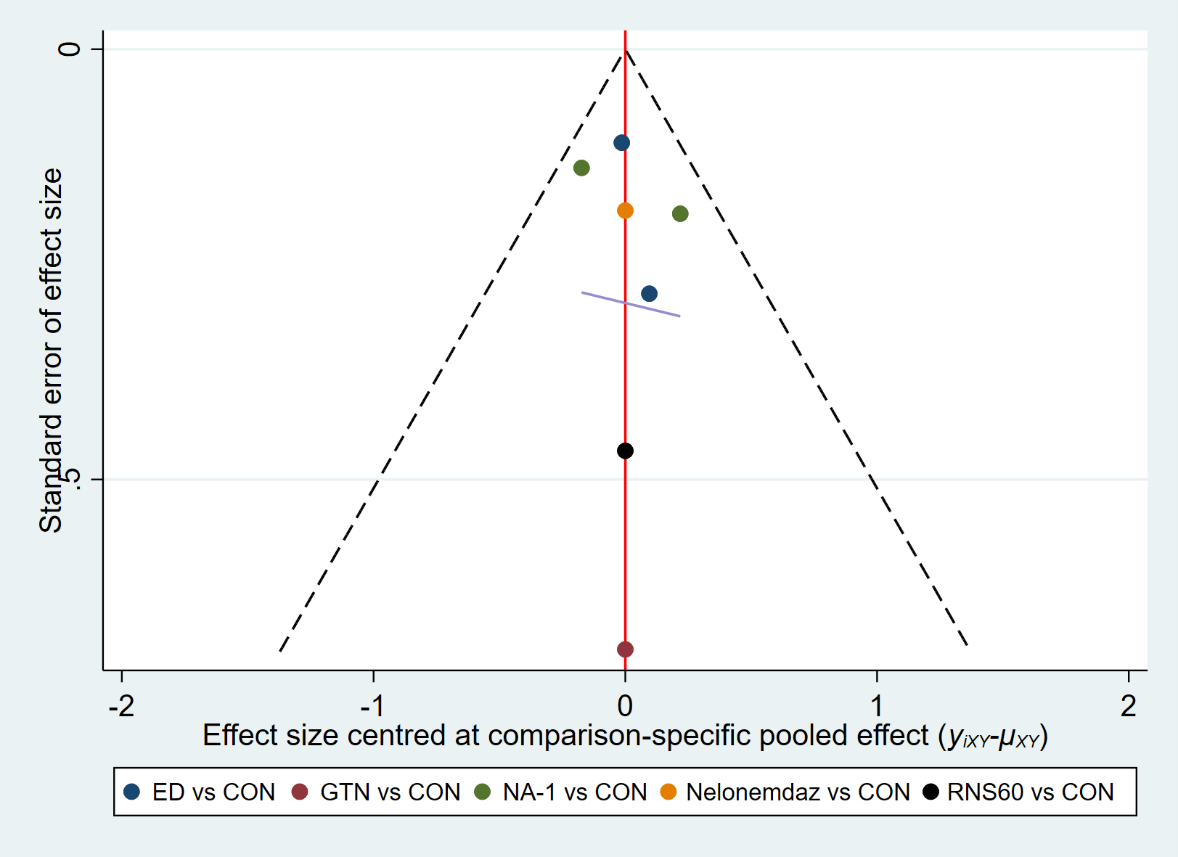


**Supplementary Figure S2.** Comparison-adjusted funnel plot for the primary outcome (functional independence at 90 days). The solid vertical line represents the symmetry axis. Visual assessment for publication bias should be interpreted with caution, as formal asymmetry testing was omitted per Cochrane guidelines due to the limited number of included trials (n = 9).


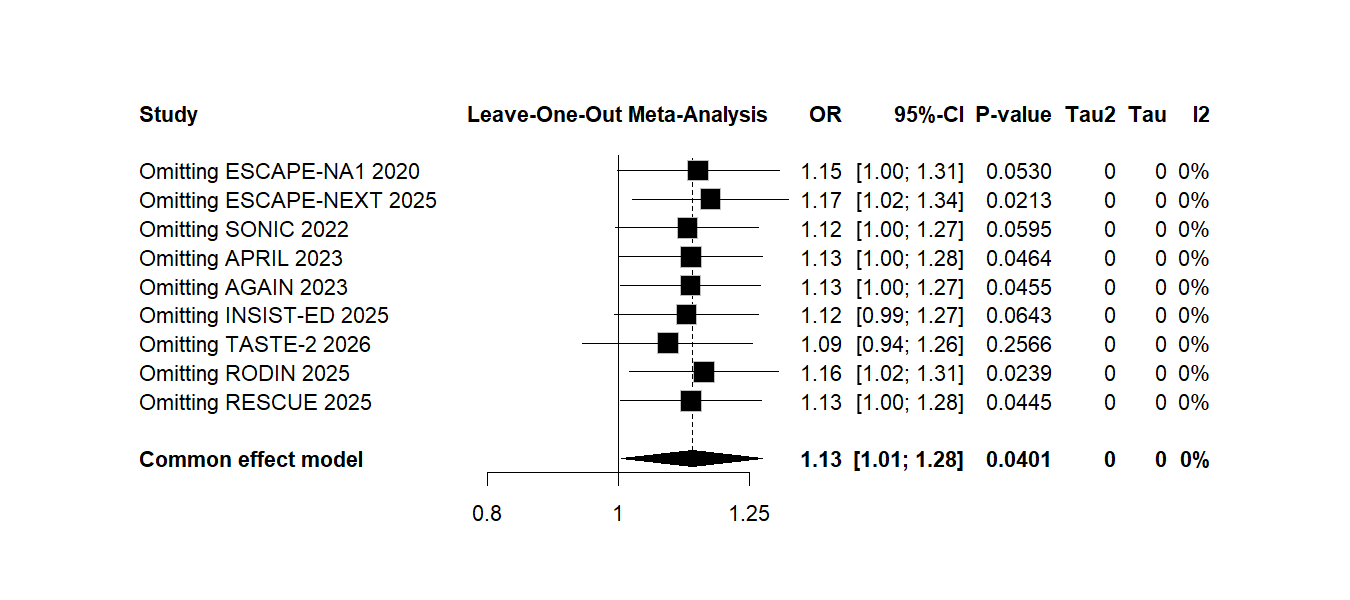


**Supplementary Figure S3.** Sensitivity analysis of the primary outcome (mRS 0–2 at 90 days). Sequential exclusion of individual studies was performed to evaluate the stability of the pooled effect estimate.


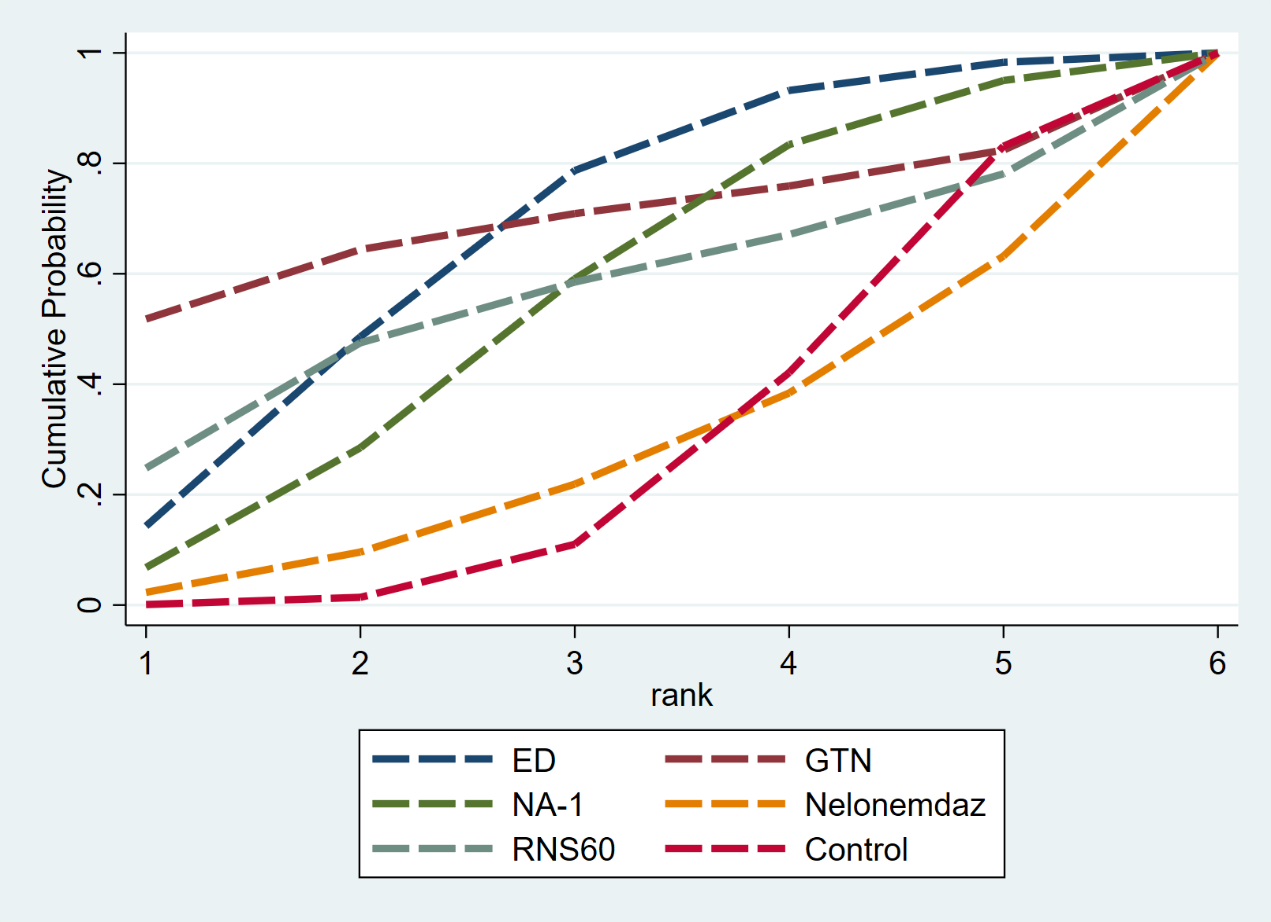


**Supplementary Figure S4.** Cumulative ranking probability plots (SUCRA curves) for the primary outcome. The curves illustrate the cumulative probability of each intervention achieving a specific rank or better. (Abbreviations: ED, Edaravone dexborneol; GTN, Glyceryl trinitrate; NA-1, Nerinetide)

**2. Supplementary Tables**

The search strategies were developed using a combination of controlled vocabulary (MeSH or Emtree) and free-text terms, and were adapted for each database.

**Supplementary Table S1.** Detailed search strategy in PubMed.

| Search | Query |
| --- | --- |
| #1 | #1: ("Stroke"[Mesh] OR "Ischemic Stroke"[Mesh] OR "Brain Ischemia"[Mesh] OR "Cerebral Infarction"[Mesh] OR stroke[Title/Abstract] OR "ischemic stroke"[Title/Abstract] OR "acute ischemic stroke"[Title/Abstract] OR "cerebral infarction"[Title/Abstract] OR "brain infarction"[Title/Abstract] OR "brain ischemia"[Title/Abstract]) |
| #2 | ("Thrombectomy"[Mesh] OR "Endovascular Procedures"[Mesh] OR "Mechanical Thrombolysis"[Mesh] OR "endovascular therapy"[Title/Abstract] OR "endovascular treatment"[Title/Abstract] OR "mechanical thrombectomy"[Title/Abstract] OR "intra-arterial thrombectomy"[Title/Abstract] OR "thrombectomy"[Title/Abstract] OR EVT[Title/Abstract] OR "stent retriever"[Title/Abstract]) |
| #3 | ("Neuroprotective Agents"[Mesh] OR "Free Radical Scavengers"[Mesh] OR "Excitatory Amino Acid Antagonists"[Mesh] OR "neuroprotect"[Title/Abstract] OR "neuroprotection"[Title/Abstract] OR "cytoprotect"[Title/Abstract] OR "nerinetide"[Title/Abstract] OR "NA-1"[Title/Abstract] OR "edaravone"[Title/Abstract] OR "edaravone dexborneol"[Title/Abstract] OR "butylphthalide"[Title/Abstract] OR "NBP"[Title/Abstract] OR "Nelonemdaz"[Title/Abstract] OR "ApTOLL"[Title/Abstract] OR "Glyceryl Trinitrate"[Title/Abstract]) |
| #4 | #1 AND #2 AND #3 |

**Supplementary Table S2.** Search strategy for Embase.

| Search | Query |
| --- | --- |
| #1 | ('stroke'/exp OR 'ischemic stroke'/exp OR 'brain ischemia'/exp OR 'cerebral infarction'/exp OR stroke:ti,ab OR 'ischemic stroke':ti,ab OR 'acute ischemic stroke':ti,ab OR 'cerebral infarction':ti,ab OR 'brain infarction':ti,ab OR 'brain ischemia':ti,ab) |
| #2 | ('thrombectomy'/exp OR 'endovascular procedure'/exp OR 'mechanical thrombectomy'/exp OR 'mechanical thrombolysis'/exp OR 'endovascular therapy':ti,ab OR 'endovascular treatment':ti,ab OR 'mechanical thrombectomy':ti,ab OR 'intra-arterial thrombectomy':ti,ab OR thrombectomy:ti,ab OR evt:ti,ab OR 'stent retriever':ti,ab) |
| #3 | ('neuroprotective agent'/exp OR 'free radical scavenger'/exp OR 'excitatory amino acid antagonist'/exp OR neuroprotect:ti,ab OR neuroprotection:ti,ab OR cytoprotect:ti,ab OR nerinetide:ti,ab OR 'na-1':ti,ab OR edaravone:ti,ab OR ' edaravone dexborneol ':ti,ab OR butylphthalide:ti,ab OR nbp:ti,ab OR nelonemdaz:ti,ab OR aptoll:ti,ab OR 'glyceryl trinitrate':ti,ab) |
| #4 | #1 AND #2 AND #3 |

**Supplementary Table S3.** Search strategy for the Cochrane Library.

| Search | Query |
| --- | --- |
| #1 | MeSH descriptor: [Stroke] explode all trees |
| #2 | MeSH descriptor: [Ischemic Stroke] explode all trees |
| #3 | MeSH descriptor: [Brain Ischemia] explode all trees |
| #4 | MeSH descriptor: [Cerebral Infarction] explode all trees |
| #5 | Ischemia:ti,ab,kw |
| #6 | #1 OR #2 OR #3 OR #4 OR #5 |
| #7 | MeSH descriptor: [Thrombectomy] explode all trees |
| #8 | MeSH descriptor: [Endovascular Procedures] explode all trees |
| #9 | MeSH descriptor: [Mechanical Thrombolysis] explode all trees |
| #10 | (endovascular therapy):ti,ab,kw OR (endovascular treatment):ti,ab,kw OR (mechanical thrombectomy):ti,ab,kw OR (intra-arterial thrombectomy):ti,ab,kw OR (thrombectomy):ti,ab,kw OR (EVT):ti,ab,kw OR (stent retriever):ti,ab,kw |
| #11 | #7 OR #8 OR #9 OR #10 |
| #12 | MeSH descriptor: [Neuroprotective Agents] explode all trees |
| #13 | MeSH descriptor: [Free Radical Scavengers] explode all trees |
| #14 | MeSH descriptor: [Excitatory Amino Acid Antagonists] explode all trees |
| #15 | (neuroprotect):ti,ab,kw OR (neuroprotection):ti,ab,kw OR (cytoprotect):ti,ab,kw OR (nerinetide):ti,ab,kw OR (NA-1):ti,ab,kw OR (edaravone):ti,ab,kw OR (edaravone dexborneol):ti,ab,kw OR (butylphthalide):ti,ab,kw OR (NBP):ti,ab,kw OR (Nelonemdaz):ti,ab,kw OR (ApTOLL):ti,ab,kw OR (Glyceryl Trinitrate):ti,ab,kw |
| #16 | #12 OR #13 OR #14 OR #15 |
| #17 | #6 AND #11 AND #16 |

**Supplementary Table S4:** Extended Search Strategies for Trial Registries and Gray Literature

| **Database / Source** | **Search Strategy** | **Notes / Filters** |
| --- | --- | --- |
| **ClinicalTrials.gov** | Condition/Disease: Stroke  Other Terms: neuroprotective AND thrombectomy  Study Type: Interventional  Status: Completed or Recruiting | Retrieved from [ClinicalTrials.gov](https://clinicaltrials.gov/) |
| **WHO ICTRP** | Search: neuroprotective AND stroke AND thrombectomy  Study Type: Interventional  Status: Completed or Recruiting | Retrieved from [WHO ICTRP](https://trialsearch.who.int/) |
| **EU Clinical Trials Register** | Search: neuroprotective AND stroke AND thrombectomy  Status: Completed or Ongoing | Retrieved from [EU CTR](https://www.clinicaltrialsregister.eu/ctr-search/search) |
| **International Stroke Conference (ISC) abstracts** | Keyword search: neuroprotective AND stroke AND thrombectomy  Year: 2018–2026 | Retrieved from [ISC abstracts](https://www.isccongress.org/search) |
| **European Stroke Organisation Conference( ESOC) abstracts** | Keyword search: neuroprotective AND stroke AND thrombectomy  Year: 2018–2026 | Retrieved from [ESO abstracts](https://www.eso-stroke.org/search/) |

**Notes for authors:**

1. All searches were conducted up to 31 January 2026, in line with the original systematic review update.
2. No additional completed RCTs meeting the predefined inclusion criteria were identified through these supplementary searches.
3. Manual screening of reference lists of included studies and relevant reviews was also performed.

**Supplementary Table S5:**

| **Unique ID** | **Study ID** | **Experimental** | **Comparator** | **Outcome** | **Weight** | **Randomization process** | **Deviations from intended interventions** | **Missing outcome data** | **Outcome measurement** | **Selection of reported result** | **Overall risk** |
| --- | --- | --- | --- | --- | --- | --- | --- | --- | --- | --- | --- |
| 1 | ESCAPE-NA1 2020 | NA-1 | Placebo | mRS 0-2 | 1 | Low | Low | Low | Low | Low | Low |
| 2 | ESCAPE-NEXT 2025 | NA-1 | Placebo | mRS 0-2 | 1 | Low | Low | Low | Low | Low | Low |
| 3 | SONIC 2022 | Nelonemdaz | Placebo | mRS 0-2 | 1 | Low | Low | Low | Low | Low | Low |
| 4 | RODIN 2025 | Nelonemdaz | Placebo | mRS 0-2 | 1 | Low | Low | Low | Low | Low | Low |
| 5 | APRIL 2023 | ApTOLL | Placebo | mRS 0-2 | 1 | Low | Low | Low | Low | Low | Low |
| 6 | AGAIN 2023 | GTN | Placebo | mRS 0-2 | 1 | Some concerns | Some concerns | Low | Low | Some concerns | Some concerns |
| 7 | RESCUE 2025 | RNS60 | Placebo | mRS 0-2 | 1 | Low | Low | Low | Low | Low | Low |
| 8 | INSIST-ED 2025 | ED | Placebo | mRS 0-2 | 1 | Low | Some concerns | Low | Low | Some concerns | Some concerns |
| 9 | TASTE-2 2026 | ED | Placebo | mRS 0-2 | 1 | Low | Low | Low | Low | Low | Low |
